# The Success of the Horse-Chestnut Leaf-Miner, *Cameraria ohridella*, in the UK Revealed with Hypothesis-Led Citizen Science

**DOI:** 10.1371/journal.pone.0086226

**Published:** 2014-01-22

**Authors:** Michael J. O. Pocock, Darren M. Evans

**Affiliations:** 1 Centre for Ecology & Hydrology, Wallingford, Oxfordshire, United Kingdom; 2 School of Biological, Biomedical and Environmental Sciences, University of Hull, Hull, United Kingdom; University Copenhagen, Denmark

## Abstract

Citizen science is an increasingly popular way of undertaking research and simultaneously engaging people with science. However, most emphasis of citizen science in environmental science is on long-term monitoring. Here, we demonstrate the opportunities provided by short-term hypothesis-led citizen science. In 2010, we ran the ‘Conker Tree Science’ project, in which over 3500 people in Great Britain provided data at a national scale of an insect (horse-chestnut leaf-mining moth, *Cameraria ohridella*) undergoing rapid range-expansion. We addressed two hypotheses, and found that (1) the levels of damage caused to leaves of the horse-chestnut tree, *Aesculus hippocastanum*, and (2) the level of attack by parasitoids of *C. ohridella* larvae were both greatest where *C. ohridella* had been present the longest. Specifically there was a rapid rise in leaf damage during the first three years that *C. ohridella* was present and only a slight rise thereafter, while estimated rates of parasitism (an index of true rates of parasitism) increased from 1.6 to 5.9% when the time *C. ohridella* had been present in a location increased from 3 to 6 years. We suggest that this increase is due to recruitment of native generalist parasitoids, rather than the adaptation or host-tracking of more specialized parasitoids, as appears to have occurred elsewhere in Europe. Most data collected by participants were accurate, but the counts of parasitoids from participants showed lower concordance with the counts from experts. We statistically modeled this bias and propagated this through our analyses. Bias-corrected estimates of parasitism were lower than those from the raw data, but the trends were similar in magnitude and significance. With appropriate checks for data quality, and statistically correcting for biases where necessary, hypothesis-led citizen science is a potentially powerful tool for carrying out scientific research across large spatial scales while simultaneously engaging many people with science.

## Introduction

In our current age, better engagement between scientists and the public is essential. One potentially effective form of engagement is to directly involve people in the scientific endeavor, which is called ‘citizen science’ [1]–[3]. Citizen science has dramatically increased in popularity in recent years. Despite the diversity of citizen science projects, the historical focus of citizen science is often on long-term, large-scale monitoring projects [3], [4]. Such large-scale projects are beyond the reach of many individual professional researchers because of the large investment required in supporting participants over many years. However, one rich, but largely untapped, role for citizen science is in participatory, hypothesis-led research [1]. Such projects clearly focus on specific hypotheses (thus being similar to much grant-led academic research) but testing hypotheses with public involvement gives researchers the opportunity to address questions at larger spatial scales and with greater temporal resolution than might otherwise be feasible; thus emphasizing citizen science's valuable role as a research tool [3], with the additional benefits of participatory engagement of the public with science.

Here, we undertook hypothesis-led research on an insect, *Cameraria ohridella* Deschka & Dimić (the horse-chestnut leaf-miner; Lepidoptera: Gracillariidae), at a national scale, in order to identify characteristics associated with its success at the margin of its expanding range. *C. ohridella* is an moth species, widely regarded as invasive, whose larvae mine the leaves of Europe's horse-chestnut *Aesculus hippocastanum* L. trees [5]–[7]. It causes striking damage to the leaves, due to the sheer abundance of the larvae, and often causes the leaves to turn brown by mid-summer. *C. ohridella* is all the more striking because it has spread rapidly throughout Europe from the Balkans over 30 years, having spread through England and Wales since its arrival in London in 2002 [8], [9]. Once established in a locality it is ubiquitous on the majority of, if not all, *A. hippocastanum* trees [10], [11].

In this project we tested specifically whether (1) levels of damage to the leaves of its host plant and (2) levels of parasitism by naturally-occurring parasitoid wasps were highest where *C. ohridella* had been present the longest. Throughout the project we undertook the essential step of data validation in order to test the accuracy of citizen science data [12]. Where data were inaccurate, we modeled the bias and propagated the uncertainty in our analyses.

For the first hypothesis our expectation was that the longer that *C. ohridella* had been present, the greater would be the amount of leaf damage. Annual increases in damage to horse-chestnut trees have been anecdotally observed e.g. [13], and quantified at small scales [10], [11], [14], but not previously quantified at large spatial scales. Here we aimed to test this hypothesis across Britain, and so quantify the shape of the relationship of damage with time.

For the second hypothesis our expectation was that the longer that *C. ohridella* had been present, the greater would be the degree of parasitism by naturally-occurring parasitoid wasps. Potentially, parasitoids could act as biocontrol agents in regulating populations of *C. ohridella* [15]. Research has been carried out previously on the parasitoids of *C. ohridella*, with several studies sampling parasitoids for one to five years at individual locations e.g. [16]–[19], and one sampling *C. ohridella* parasitoids across Europe from sites where *C. ohridella* had been present for one to 20 years [20]. In the current study, volunteers reared parasitoids from *C. ohridella* from sites across Britain in order to assess the parasitism of *C. ohridella* early in its establishment in a country.

## Materials and Methods

### Estimating the relationship of A. hippocastanum leaf damage with the length of time that *C. ohridella* had been present

**Figure 1:**
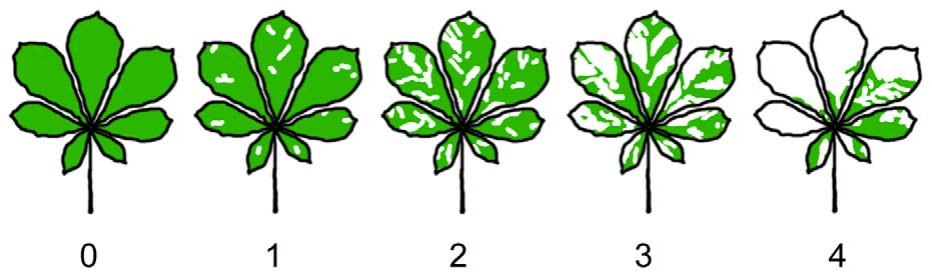
Scoring damage to horse-chestnut leaves. Diagrams of leaf damage given to participants to score damage to horse-chestnut *Aesculus hippocastanum* leaves, adapted from [12]. The accompanying descriptions were: 0 = The leaf is completely green. No evidence of moth attack; 1 = Just a couple of whitish/brown patches on the leaf; 2 = There is more green than white/brown on the leaf; 3 = You can't decide whether green or white/brown dominates. It's about half green leaf and half whitish/brown patches; 4 = The whitish/brown patches definitely cover more than half the leaf. They may cover all the leaf.

During 2010, we ran a hypothesis-led citizen science project (called ‘Conker Tree Science’) to study the biology of *C. ohridella*. In order to quantify the relationship of the amount of damage to the leaves of *A. hippocastanum* with the length of time that *C. ohridella* had been present, participants recorded leaf damage on horse-chestnut trees on an ordinal scale (0–4) during the summer (15 Jun to 30 Sep 2010). The scoring was undertaken with a simplified version of the scoring in [12] (Fig. 1). Scores were reported via forms on a purpose-built website (http://www.conkertreescience.org.uk). In order to assess the accuracy of these scores, we obtained photos of 4882 leaves (from 2153 participants; uploaded via a smart phone app as part of a different project; see http://leafwatch.naturelocator.org/) which had been scored by the participant. We tested the concordance between participants' scores and those from an experienced recorder to confirm that leaf damage was scored accurately. To assess concordance we used the quadratically-weighted kappa statistic, which is a chance-corrected measure of absolute agreement suitable for ordinal data [21].

Where participants reported damage (score>0), we used ordinal regression [22] to relate the reported damage score to the number of years that *C. ohridella* was predicted to have been present at that location and the number of days since 1^st^ June (about the time of year when leaf mines first become evident in Britain). By only considering trees that had reported damage we avoided the problem of needing to exclude non-susceptible *Aesculus* species, such as *A. carnea* J. Zeyh. [9]. We ran candidate models in which there was a linear, quadratic, log-linear and segmented regression of damage against the number of years present. For the segmented regression, we considered models in which there was a positive linear or log-linear relationship up to a break-point value (of between 2 and 7 years inclusive, depending on the model), after which the relationship was constant. Segmented regression models were a tractable way to approximate inter-annual logistic growth of the moth with ordinal regression, i.e. to model annual damage increasing rapidly up to a maximum representing the carrying capacity, as would be expected for a species expanding into an unoccupied niche. We assessed the relative fit of the models based on Akaike's Information Criterion, and we undertook model averaging based on Akaike weight [23] in order to estimate the overall annual damage, i.e. the predicted damage at the end of September.

### Estimating the true spread of *C. ohridella*

One important source of potential bias that we needed to consider was the apparent under-recording of *C. ohridella*'s distribution in Britain in recent years. The year that *C. ohridella* had first been detected in a locality (specifically a 10×10 km square) since its arrival in Britain in 2002 was collated by Forest Research [24]. Once *C. ohridella* had spread widely in Britain (specifically after 2006), the maps collated by Forest Research suggest that the area of occupancy of the moth has not increased as quickly as expected. Specifically, the plot of annual range size against annual proportional increase in range size is unexpectedly variable after 2006; so the low figure for 2007 combined with the spatial patchiness of the maps of distribution suggests substantial under-recording of the true distribution (Fig. S1). If targeted search effort had continued we would have expected a more consistent range-filling spread of the moth, as has occurred with other range-expanding leaf-mining moths on non-native plants in Britain [25]. We anticipated that under-recording would occur as *C. ohridella* became established because the search area becomes larger and national interest in the species is reduced (the species is becoming ubiquitous, rather than being novel). This is especially so in presence-only data where recorder effort is not known, as in this case.

**Figure 2:**
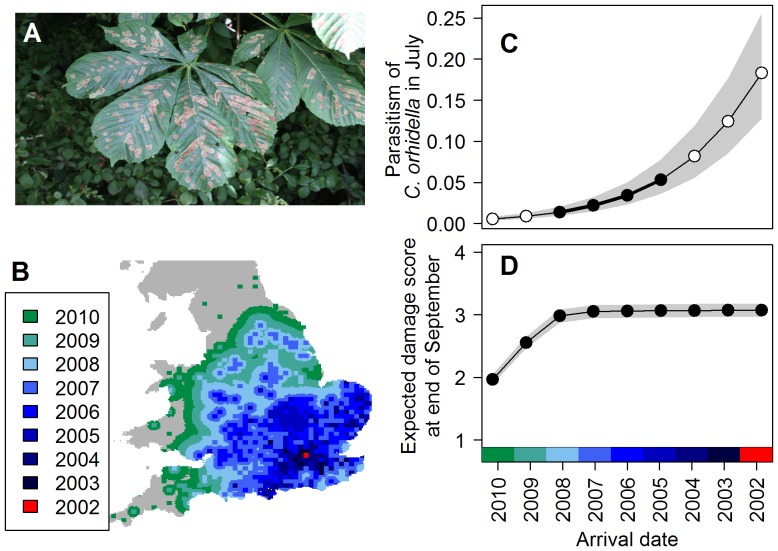
The spread of the leaf-mining moth and how the length of time it has been present in a location affects leaf damage and the level of attack by parasitoids. (A) The larvae of the leaf-mining moth *Cameraria ohridella* cause distinctive damage to horse-chestnut leaves. (B) The modeled distribution of the spread of *C. ohridella* produced by augmenting the observed distribution after 2006 with a demographic model of spread assuming two generations per year. (C) The effect of the length of time that *C. ohridella* has been present on levels of attack by parasitoids (‘parasitism’) as estimated from the data (black points) and extrapolated from the model (white points) with the inter-quartile range (grey). (D) The effect on leaf damage of the length of time that *C. ohridella* has been present, based on expected results from an ordinal regression, with inter-quartile range (grey).

In order to overcome the potential under-recording of *C. ohridella*, we used a demographic model to predict the arrival time of *C. ohridella* in each 10×10 km square. Specifically, we did this by augmenting its recorded distribution each year from 2006 to 2010 with a demographic model of spread that had been previously parameterized from continental Europe [6], [8], hereafter referred to as the ‘modeled distribution’ (Fig. 2B and Fig. S1). This demographic model of spread is probabilistic and it comprised two components: one represented diffusion over short distances and one represented rare long-distance (probably human-assisted) movements. For our modeled distribution we used the short-distance dispersal parameter from the published demographic model [6] to model diffusive spread (probability of presence = exp(−0.025×*d* ^2^), where *d* is distance to the nearest occupied square in km). We did not use the long-distance dispersal parameter in our modeled distribution, but instead we allowed the data themselves to inform us of these long-distance dispersal events causing extra-limit range expansion. We ran the models on a 2.5 km grid to replicate the original models [8]. Although *C. ohridella* typically has three generations per year in much of its range, in southern England it has two generations and a partial third generation [11]. We therefore ran the model twice, once with two and once with three generations per year. We began modeling the distribution from the start of 2006. At the end of every second or third generation (which, depending on the model, was equivalent to the end of 2007, 2008, 2009 and 2010) we updated the predicted distribution with the observed distribution at the end of that year. When updating the distribution, we assumed that the moth was present in all sixteen of the 2.5 km grid cells in each of the 10 km grid cells that it had been recorded as present; an assumption, which given the rapid rate of spread of *C. ohridella*, would have a relatively small effect on the overall distribution. The dispersal model was probabilistic, so we calculated the median year of arrival in each 10 km square across the 99 model runs.

### Estimating the relationship of parasitism with the length of time that *C. ohridella* had been present

In order to assess the levels of parasitism of larvae of *C. ohridella*, we invited people to rear insects from horse chestnut leaves infested with *C. ohridella*. Participants sampled leaves during the first week of July 2010 (i.e. the first of the moth's generations that year) and stored them in sealed plastic bags for two weeks. We then asked participants to report the number of leaf-mines, and to identify and count the insects in each category: adult *C. ohridella* moths, parasitoids, and ‘other insects’. These counts were reported via forms on a purpose-built website (http://www.conkertreescience.org.uk). We included the ‘other insect’ category as a catch-all for all items apart from those in which we were specifically interested. Therefore this would have included insects accidentally introduced to the sample bags and may have included *C. ohridella* caterpillars exiting their leaf mine or empty pupal cases. Counts in the ‘other insect’ category were not considered for analysis. Anyone could take part in rearing parasitoids, but we particularly focused on school children aged 8–11 by working with a team of eight trained volunteers across the country (Fig. S2) who directly contacted schools and led lessons in classes. Each volunteer worked with between 3 and 15 classes (median = 9) of up to 32 children each. When visiting schools, the volunteers provided the children with general guidance about how to collect and store leaves and identify emergent insects. They did not provide directive guidance during the time that the children were counting adult moths and parasitoids, so the data were not biased by our supervision.

At the completion of the activity and after the children had recorded their results, we retained a randomly-selected subset of 669 samples that the children had counted. For these samples we used quadratically-weighted kappa [21] to assess concordance between the children's and the expert's count of leaf mines and adult moths (to check the accuracy of the children's counts). We also retained an additional 75 samples in which children had reported parasitoids. We tested the concordance between children's and the expert's count for all samples in which children had reported parasitoids (n = 187) and assessed the presence of all ‘other insects’.

We then tested the relationship of parasitism with the number of years that *C. ohridella* had been present in a location. We defined parasitism as number of parasitoids/(number of parasitoids plus *C. ohridella* adults; see Methods S1 for discussion of this metric). The rate of parasitism was therefore estimated at the level of the sample (the leaf) rather than the more precise approach of estimating at the level of the mine or pupae [16]–[20]. Although this metric was an index of the rate of parasitism (rather than being an estimate of the ‘true’ rate), it was used consistently across sites and so was sufficient for testing our hypothesis, but it meant that in was inappropriate to conduct a direct comparison of our index with estimates of parasitism derived from other approaches. We included random effects to account for systematic variation between individuals and groups (specifically: for school classes the random effects were class nested within the volunteer, with volunteers each visiting multiple classes; for other participating members of the public the random effect was the individual). We only considered data from 10 km squares within which *C. ohridella* was predicted to have been present for 3, 4, 5 or 6 years, because sample sizes were too small (<19 for each year) otherwise. We also ran a full model in which we included the number of mines in the leaf and location (easting and northing at the 10 km scale) as covariates.

**Figure 3:**
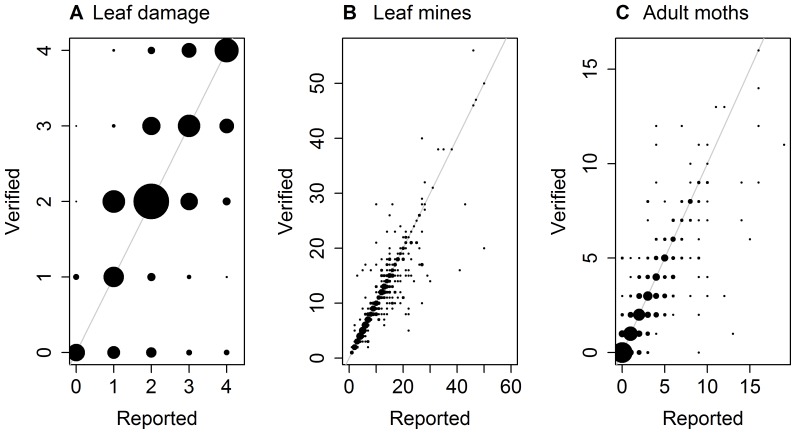
Accuracy of reported counts of (A) leaf damage, (B) leaf mines and (C) adult moths. The area of the circles is proportional to the frequency of reports. The maximum circle size represents 1233, 20 and 178 records for A, B and C, respectively. The 1∶1 line is shown in grey.

In the subset of retained samples we found high concordance between the children's and the expert's counts for most variables, so they could be used in the analysis without correction (see Results). However, counts of parasitoids showed very low concordance (Table 1). We therefore needed to (1) quantify this uncertainty and (2) propagate the uncertainty caused by this bias into the final analysis. In order to quantify the error, we modeled the relationship between the children's counts of parasitoids (where they were greater than zero) and the expert's counts using a zero-inflated Poisson regression. Where the children's counts were equal to zero, we directly calculated the probability distribution of the true number of parasitoids. In order to propagate the uncertainty, we bootstrapped the parasitoid count data from the relationship with the true counts (Fig. 3) and ran the logistic regression described above, repeating this 100 times. We estimated the final parameter estimates and their variance with multiple imputation [26]. In summary, although this approach seems complex, we are confident that it was the best way to use the biased citizen science parasitoid count data to produce statistically-robust results.

**Table 1:** Concordance (quadratically-weighted kappa) between the citizen science and expert-validated data based either on submitted photographs (leaf damage scores) or a retained subset of samples (all other records).

Data	N	Quadratically-weighted kappa
Leaf damage scores from submitted photographs	4882	0.754
Counts of reared adult *C. ohridella* moths	643	0.822
Counts of reared parasitoids*	187	0.068
Counts of *C. ohridella* leaf mines	484	0.720

Kappa varies from zero (no concordance) to one (perfect concordance).

The vast majority of samples had no parasitoids, so we only assessed the concordance for samples in which a parasitoid was reported.

All data are freely available via doi:10.5285/9f913f10-6e3d-449e-b8af-8fa2d06d7fd3.

## Results

In total, over 3500 people were engaged with the Conker Tree Science project in 2010. Over 4000 records were entered from across the current distribution of *C. ohridella* (Fig. S2), allowing us to address two scientific hypotheses at a national scale.

We considered demographic models of spread to take account of potential under-recording of the distribution of *C. ohridella*. We found that the number of years that *C. ohridella* had been present estimated from the modeled distribution assuming two generations per year (Fig. 2B) explained the data better than assuming three generations per year. Specifically, it was ranked highest in the set of models of the leaf damage data (Table 2) and it showed a more constant decline in the proportional area of increase over time than either the observed year of arrival or the modeled distribution assuming three generations (Fig. S1).

**Table 2:** The top-ranked set of ordinal regression models relating leaf damage of *A. hippocastanum* with the number of years that *C. ohridella* was predicted to have been present.

Model type*	Number of *C. ohridella*	Break-point	ΔAIC	AIC
	generations to model distribution†	(years)		weight
Log-linear segmented	2-generation model	3	0	0.395
Linear segmented	2-generation model	3	0.4	0.319
Linear segmented	3-generation model	4	2.7	0.105
Log-linear segmented	2-generation model	4	4.0	0.053
Log-linear segmented	3-generation model	4	4.7	0.038
Log-linear segmented	2-generation model	7	6.1	0.019
Log-linear segmented	2-generation model	n/a	6.2	0.018
Log-linear segmented	2-generation model	6	6.9	0.013
Log-linear segmented	2-generation model	2	8.5	0.006
Linear segmented	2-generation model	2	8.5	0.006
Log-linear segmented	3-generation model	7	8.6	0.005
Linear	3-generation model	n/a	9.0	0.004
Log-linear	3-generation model	n/a	9.0	0.004
Log-linear segmented	3-generation model	6	9.1	0.004
Log-linear segmented	2-generation model	5	9.5	0.003
Linear segmented	2-generation model	4	9.6	0.003

Models are ordered by relative model fit (ΔAIC) and only those with ΔAIC<10 are shown here.

The form of the relationship of years present with leaf damage. The segmented regression models show a linear or log-linear relationship up to the break-point, after which the relationship is constant.

All the top-ranked regression models were based on the modeled distribution of *C. ohridella*, assuming either 2 or 3 generations per year. Regression models including the directly observed year of arrival were included in the candidate model set, but all had ΔAIC>10.7, so are not shown here.

In total, we received 1823 records of *A. hippocastanum* leaf damage from 875 participants. Expert scoring of 4882 photographs submitted for a separate project, but with the same protocol as this project, showed that there was high concordance between participants and an expert's scores, suggesting that these citizen science data were accurate (Table 1; Fig. 3A).

The best ordinal regression model of the relationship of leaf damage with the number of years that *C. ohridella* had been present was the segmented regression in which there was a linear increase in damage for the first three years (having taken account of the time of year and using number of years that the moth had been present based on the modeled distribution assuming two generations per year), with constant levels of damage thereafter (Table 2). The summed AIC weights of models with the modeled distribution assuming two generations was 0.839, while the summed weights for models with the modeled distribution assuming three generations and for the observed distribution were 0.161 and <0.001, respectively. The summed AIC weights for segmented regression models with a break point at three and four years was 0.717 and 0.200, respectively. These values can be interpreted as the proportional support for these model parameters, showing there was most support for the distribution spread from 2006 to 2010 when modeled with two generations, and most support for models with a break point at three years, though some support for those with a break point at four years. The model-averaged results of the models for expected (mean) leaf damage at the end of September show negligible increases in damage after the first three to four years (Fig. 2D). Adding location (eastings and northings) to the best ranked model showed that both of these were important (Table S1). However, interpretation of these results is complicated by the fact that *C. ohridella* spread from south-east England, so eastings and northings are confounded with the number of years that the moth had been present. Therefore although geographic position could have affect the observed mine densities, we could not distinguish this from the effect of the number of years on levels of infestation.

**Figure 4:**
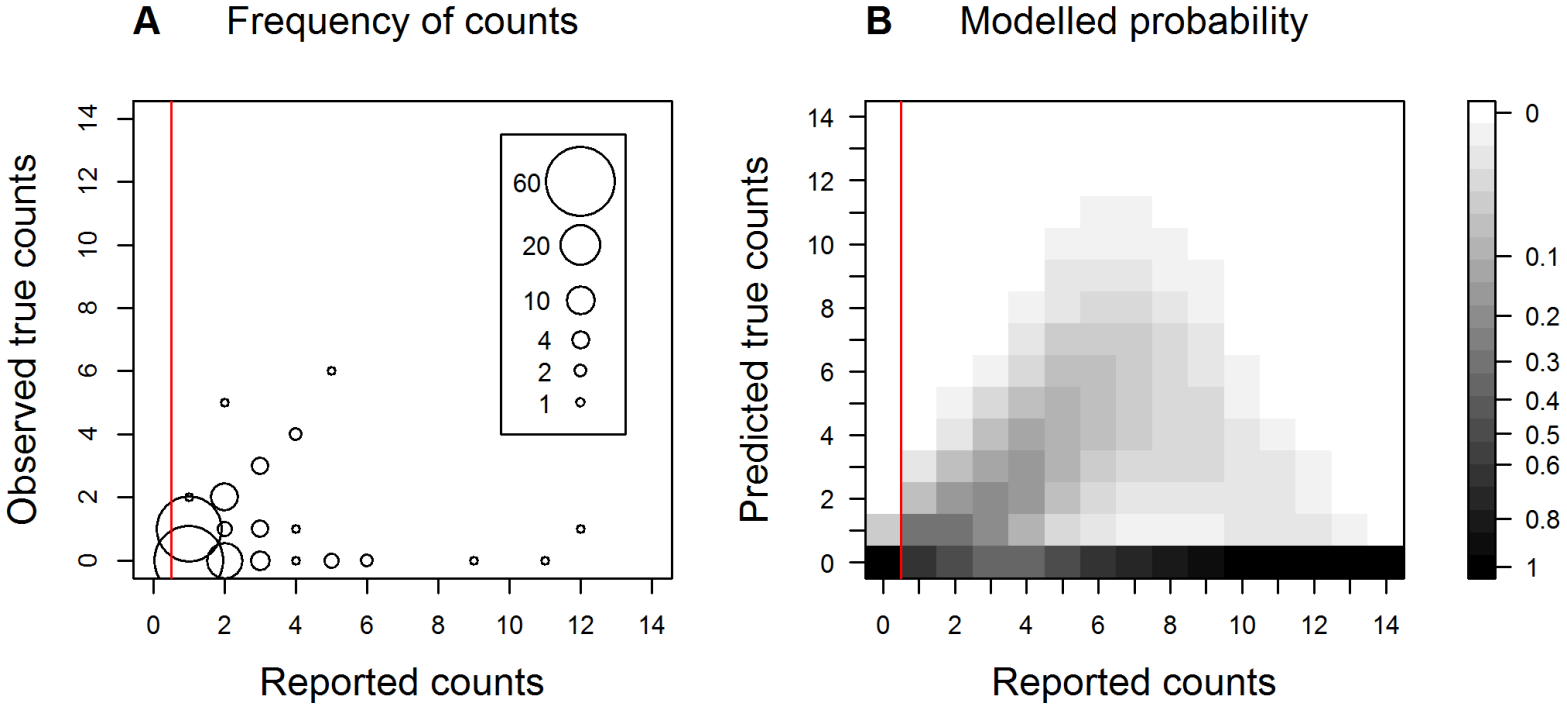
Quantified bias of the citizen science data of counts of parasitoids. (A) The observed frequency of the true number of parasitoids against the reported number, which was determined from samples retained from participating school children. Frequencies are not shown when the reported number of parasitoids was equal to zero (to the left of the red vertical line). (B) The probability distribution of the true number of parasitoids against the reported number, based on a zero-inflated Poisson regression of a subset of validated records. To the left of the red vertical line the probabilities were directly calculated, while to the right they were estimated from the model.

We received 2208 records of reared insects, in total, from 2059 people (1810 school children and 398 records from 119 members of the public). From the retained subsets, we found that adults and larval leaf mines of *C. ohridella* were counted accurately, but that parasitoids were counted less accurately (Table 1; Fig. 3B–C, Fig. 4A). In these retained samples, we found 14% of samples contained one or more parasitoids and only 7% contained an insect that was not *C. ohridella* (which in almost all cases was a single Psocopteran or aphid, both of which were illustrated on our project's identification guide). From our data we directly calculated that when the reported number of parasitoids was zero, the probability that there were 0, 1, 2 or >2 parasitoids was 0.96, 0.04, <0.01 and 0%, respectively. When considering the relationship between the recorded and the true number of parasitoids (where recorded numbers were greater than zero), the best zero-inflated Poisson regression model was one that had a linear binomial component and a quadratic Poisson component (Table 3 and Fig. 4B). One interpretation of this is that people seeing parasitoids accurately reported the number of parasitoids, while others reported parasitoids (sometimes many parasitoids) when there were none (Fig. 4). We used the zero-inflated Poisson model results to propagate a correction for bias into our final analysis (as described in the Methods).

**Table 3:** The candidate set of zero-inflated Poisson regression models of the relationship of true counts with reported counts of reared parasitoids.

Form of the binomial component	Form of the Poisson component	ΔAIC
Linear	Quadratic	0
Constant	Quadratic	8.25
Linear	Linear	21.08
Constant	Linear	24.27
Constant	Constant	27.50
Linear	Constant	29.32

We found that the longer that *C. ohridella* had been present in a location in Britain, the higher was the rate of parasitism (Table 4; Fig. 2C; P<0.012, with variation in the degree of significance depending on the number of generations used in the modeled distribution). We discovered that when we used the bias-corrected data it led to a reduction in the estimated rate of parasitism (e.g. estimates when the moth had been present for six years, i.e. in 2005, were 5% with the bias-corrected data and 9% with the raw data), but the relationship was significant for both (for the modeled distribution assuming two generations per year P = 0.012 with the bias-corrected data and P = 0.004 with the raw data; Table S2). In the full model, the effect of location (eastings and northings) was not significant, but increasing the number of mines had a weakly significant negative effect on parasitism (Table 4; Fig. S3). This could be explained by pro-rata dilution of the impact of parasitoids with increasing host density. Overall, our findings of the effect of the length of time that *C. ohridella* had been present were consistent and robust to the specific method adopted (Methods S1; Fig. S4).

**Table 4:** The parameter estimates (excluding random effects) from the logistic generalized linear mixed model predicting the effect of length of time since arrival on parasitism.

	Modeled distribution of *C. ohridella* assuming two generations per year			Modeled distribution of *C. ohridella* assuming three generations per year		
	Coefficient	SE	P	Coefficient	SE	P
Simple model						
Intercept	−5.490	0.763	<0.001	−5.531	0.754	<0.001
Years since arrival	0.430	0.163	0.012	0.439	0.161	0.009
Full model						
Intercept	−4.221	1.159	<0.001	−4.156	1.164	<0.001
Years since arrival	0.379	0.178	0.004	0.356	0.178	0.005
Easting (GB grid ref in km)	−0.001	0.003	0.362	−0.001	0.003	0.372
Northing (GB grid ref in km)	−0.001	0.002	0.377	−0.001	0.002	0.359
Number of mines	−0.010	0.004	0.038	−0.009	0.004	0.040

## Discussion

### Increasing our understanding of the success of *C. ohridella*

Citizen science allowed us to undertake data collection over a large spatial extent. The data allowed us to estimate that it takes just three to four years for the moth to cause maximum levels of damage (Fig. 2D). This supports previous anecdotal statements [12] and single-site studies [10], [11], [13], but this was the first time this has been quantified at such a large scale. Our segmented regression of the ordinal data of leaf damage approximated to an annual logistic growth model for *C. ohridella* as would be expected for a species invading an empty niche. However, given the observed spread of the moth, we could not formally distinguish the effects of time since first arrival from geographic position (eastings and northings), which could also have had an impact on the observed density of leaf mines. Our data were collected over one year, but the actual maximum level of damage will vary annually [11], [18], however we expect that the effect of the length of time the moth has been present to remain consistent.

The role of species interactions in range shifts has received increasing interest recently, e.g. [27]. Our finding was that the longer that *C. ohridella* has been present in a location, the higher the rate of parasitism (Fig. 2C). This has not previously been shown so clearly, except in a large study across continental Europe covering sites where *C. ohridella* had been present for one to 20 years [20]. That study, like the current study, also found increasing parasitism over time. In that case it was found to be due to increases in parasitoids that mainly attack the pupal stages of *C. ohridella*, e.g. *Pedobius saulius* Walker and *Closterocerus trifasciatus* Westwood, while the abundance of parasitoids that mainly attack pre-pupal stages, such as *Minotetrastichus frontalis* Nees and *Pnigalio* spp., showed no significant relationship with the length of time that *C. ohridella* had been present [20]. This finding does not seem to explain the increase in parasitism over time in Britain because the identification of a sample of parasitoids reared from *C. ohridella* across Britain [28] found that all species (with the exception of the rarely-encountered *C. trifasciatus*) were those that attacked the pre-pupal stages of *C. ohridella*. If this interpretation is correct, what does it reveal about the successful spread of *C. ohridella*? It could be explained by there being two stages to the recruitment of parasitoids by a novel host. The first stage (demonstrated by our result from the leading edge of *C. ohridella*'s range expansion) being the recruitment of generalist pre-pupal parasitoids from the local environment, as found with other leaf-miner species [29], while the second stage is a longer-term response, which might be due to particular species, or races of species, adapting to or tracking their host [30]. Our index of parasitism rates are similar to those estimated, using more precise methodology, in continental Europe [20]. In continental Europe those rates have not continued to rise indefinitely, so we expect that the rate of increase of parasitism will plateau over the next few years in Britain. This has also been demonstrated in the parasitism of other herbivorous insects [31]–[33]. In the case of *C. ohridella* the overall low rate of parasitism across its range appears to have contributed to its rapid spread and is probably due to asynchrony between the spring emergence of the parasitoids and the susceptibility of the host [34].

### Implications for citizen science

We believe that our study not only reveals new results about a rapidly spreading insect, but it also highlights the advantages and disadvantages of a citizen science approach, especially short-term hypothesis-led citizen science. This short-term approach to citizen science is not common, though there are a few excellent examples [35], [36]. Our project provided results with just one year of data collection and we were able to gather data almost daily across a whole summer and with much greater spatial coverage and finer resolution than would otherwise have been possible. This means that as well as fulfilling its objective to address two specific hypotheses, our project exemplifies the opportunities for a larger number of scientists to consider citizen science as a valuable form of research.

Citizen science is often without cost at the point of data collection, as it was in the current study, but it is not free [37]. The whole of our current study cost the equivalent of a single UK postgraduate student working for 18 weeks. If travel and subsistence costs were added to this, then it would have been prohibitively expensive to collect these data without involvement from citizen science participants. By adopting a citizen science approach we also had the benefit of stimulating public engagement with science while undertaking research, and had the benefit of developing of a re-usable web-based infrastructure to support recording in following years. An additional benefit of engaging members of the public was adding to the currently known distribution of *C. ohridella*; this recording of distribution change is a well-known and valuable aspect of ‘citizen science’, indeed such a project was undertaken for another leaf-mining moth in the UK two decades previously [38]! However, when considering the data themselves (excluding the benefits of public engagement) an alternative approach could have been to undertake structured surveying by professionals at a smaller number of carefully-selected sites in order to address the same hypotheses, and this could also have permitted the collection of many more measurements as part of the study, e.g. [11]. One important weakness of citizen science was that we were unable to obtain the parasitoid specimens. If we had these specimens, we could have identified them, so allowing us to test our supposition about the composition of parasitoid species in Britain compared to continental Europe. Citizen science therefore provides advantages and disadvantages as a data-collection tool: it is not the universal answer for data collection, but its advantages may make it very appealing for addressing particular questions.

One of the big challenges with reporting results of citizen science is the need to demonstrate the trustworthiness of the data. Often the data quality is excellent [11], but this cannot be assumed and citizen science data, like any data, need appropriate validation [39], [40]. Usually validation of citizen science data can only take place after the data have been collected, whereas for data collected by ‘professional’ scientists the quality control is usually in advance of data collection via training. To validate all citizen science data after submission can be time consuming and can induce bias, depending to the method of validation [41]. In our case it was completely infeasible to request that people submitted photographs of parasitoids <1 mm long for validation, and it would have reduced the number of people involved if we had required that parasitoid specimens were submitted. Therefore we validated a subset of the data, modeled the bias and carried this uncertainty through the final analysis. Though by no means unique in scientific research, it is uncommon to propagate ‘measurement uncertainty’ through analyses of empirically-collected citizen science data. However, we note that most of our protocols allowed participants to record data accurately (Table 1) which, when possible, is a much better strategy than subsequently coping with biased data [42]. Recorded counts of parasitoids were less accurate than other counts. Our anecdotal experience suggests that this was related to the children's experience of parasitoids, i.e. it appeared that some of those that did not see any parasitoids appeared to record any non-moth item as a parasitoid. Maybe the probability of encountering parasitoids (calculated to be about 14% across the samples) was too low for inexperienced participants to accurately report their presence, whereas almost everyone encountered moths (Fig. 2) resulting in much higher accuracy of counts (Table 1). This reinforces the care that must be applied for the design of excellent citizen science protocols. However, the rearing of parasitoids also demonstrates what is possible for citizen science; rearing miniscule insects is an involved procedure which we ambitiously asked people to do, but it was a task that many people undertook successfully.

Academics are increasingly aware of the importance of public engagement with science, yet are time-stretched and the pressure to deliver excellent science has never been greater. Can this predicament be resolved? We believe the answer can be yes. Our example of a focused citizen science approach allowed us to reveal novel aspects of an invading non-native insect's biology with relatively little cost, whilst simultaneously involving about 3500 public participants directly in scientific research in one year – a true partnership between research and engagement [43]. We empirically show that public engagement need not be a bolt-on to primary research, rather, in hypothesis-driven citizen science, public engagement and research are synergistic.

## Supporting Information

Figure S1 **Comparison of the observed and modeled distribution of the spread of** ***C. ohridella*** **.** The (A) directly observed distribution and the modeled distribution, based on augmenting the observed distribution with a demographic model of spread, of *C. orhidella* in Britain since 2006 when assuming *C. orhidella* has (B) two generations per year or (D) three generations per year. The comparison of the directly observed and modeled rate of spread of *C. ohridella*, when assuming *C. orhidella* has (C) two generations per year or (E) three generations per year, suggests that the increasing distribution was under-recorded since 2006, while augmenting the observed distribution with the predicted spread, based on demographic models, shows a more consistent declining trend (especially when assuming two generations per year; C) and a better fit to the damage scores (see Results). We therefore consider that substantial deviations from the observed and predicted time of arrival in years beyond 2006 (e.g. in south-east England) are largely due to under-reporting.
(TIF)

Figure S2 **The distribution of records received from Conker Tree Science participants in 2010.** (A) The distribution of records of leaf damage that were received. (B) The distribution of records of parasitoids and adult moths that were received, with diamonds indicating where volunteers worked with local schools.
(TIF)

Figure S3 **The effect of the number of mines on the estimated rate of parasitism.** The results of the simple model (Fig. 2c) are overlaid with estimates from the full model for different numbers of mines, according to their distribution in the dataset (from top to bottom): 2 mines (5^th^ percentile), 14 mines (median), 24 mines (75^th^ percentile) and 55 mines (95^th^ percentile).
(TIF)

Figure S4 **Modeled minimum number of parasitism events from reported counts of parasitoids.** This model is the repeat of Fig. S3, but with the minimum number of parasitism events as defined in Methods S1.
(TIF)

Methods S1 **Measuring parasitism and taking account of uncertainty in the count data.**
(DOCX)

Table S1 **Results of the best model from** **Table 2** **(log-linear segmented regression with the break point being at two years and assuming a modeled distribution with two generations per year) with additional effects of location (eastings and northings).**
(DOCX)

Table S2 **Effect size of the length of time that the** ***C. orhidella*** **had been present when considering different numerators and denominators in the definition of parasitism, and whether the data were adjusted to correct for bias in the reported data.**
(DOCX)
