# Supplementary figures and images for: The Success of the Horse-Chestnut Leaf-Miner, Cameraria ohridella, in the UK Revealed with Hypothesis-Led Citizen Science
Source: PLoS One. 2014 Jan 22;9(1):e86226. doi: 10.1371/journal.pone.0086226 (PMC3899221; doi:10.1371/journal.pone.0086226)

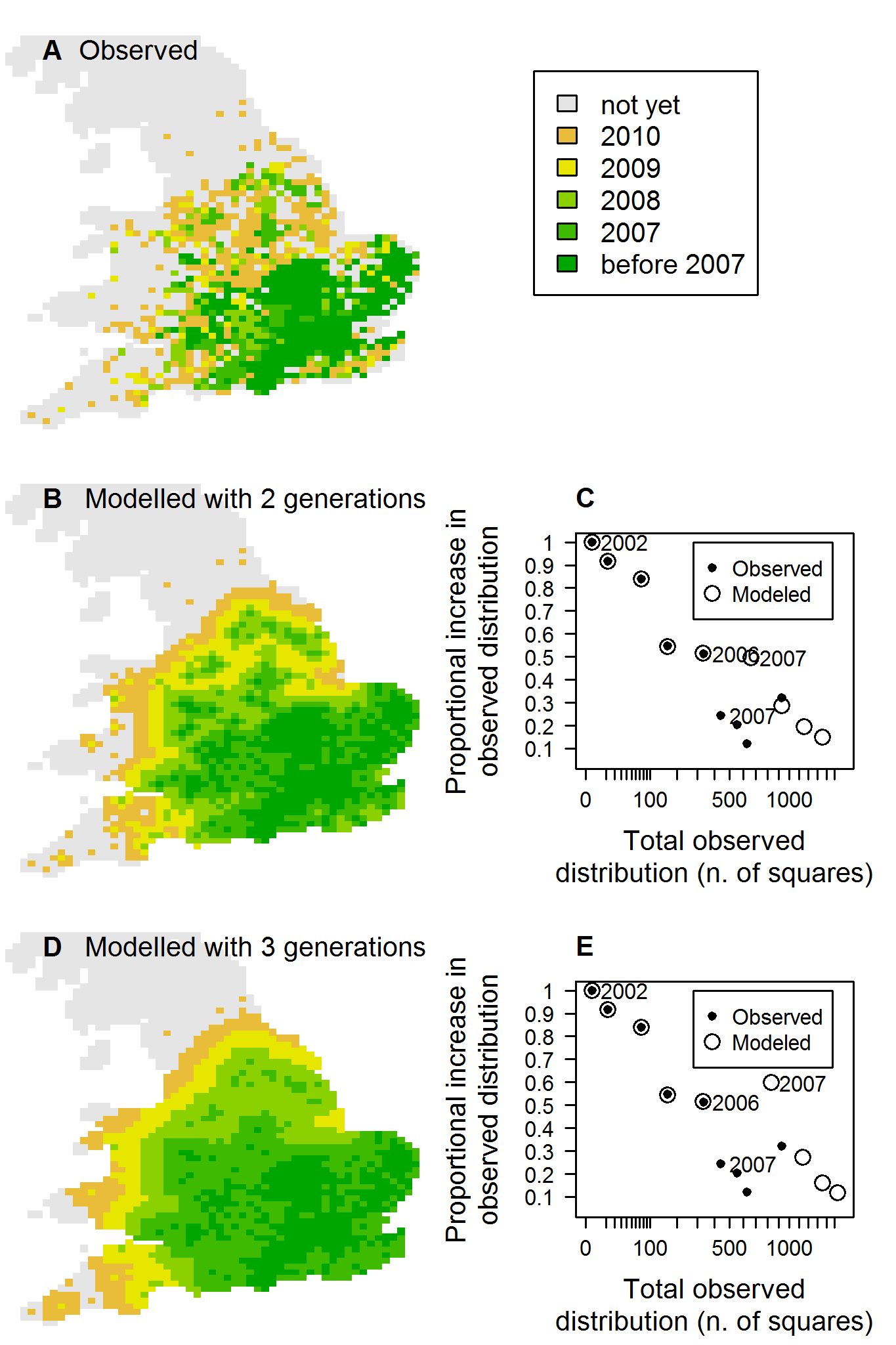

Supplement: Figure S1 — Comparison of the observed and modeled distribution of the spread of C. ohridella . The (A) directly observed distribution and the modeled distribution, based on augmenting the observed distribution with a demographic model of spread, of C. orhidella in Britain since 2006 when assuming C. orhidella has (B) two generations per year or (D) three generations per year. The comparison of the directly observed and modeled rate of spread of C. ohridella, when assuming C. orhidella has (C) two generations per year or (E) three generations per year, suggests that the increasing distribution was under-recorded since 2006, while augmenting the observed distribution with the predicted spread, based on demographic models, shows a more consistent declining trend (especially when assuming two generations per year; C) and a better fit to the damage scores (see Results). We therefore consider that substantial deviations from the observed and predicted time of arrival in years beyond 2006 (e.g. in south-east England) are largely due to under-reporting. (TIF) [file pone.0086226.s001.tif]

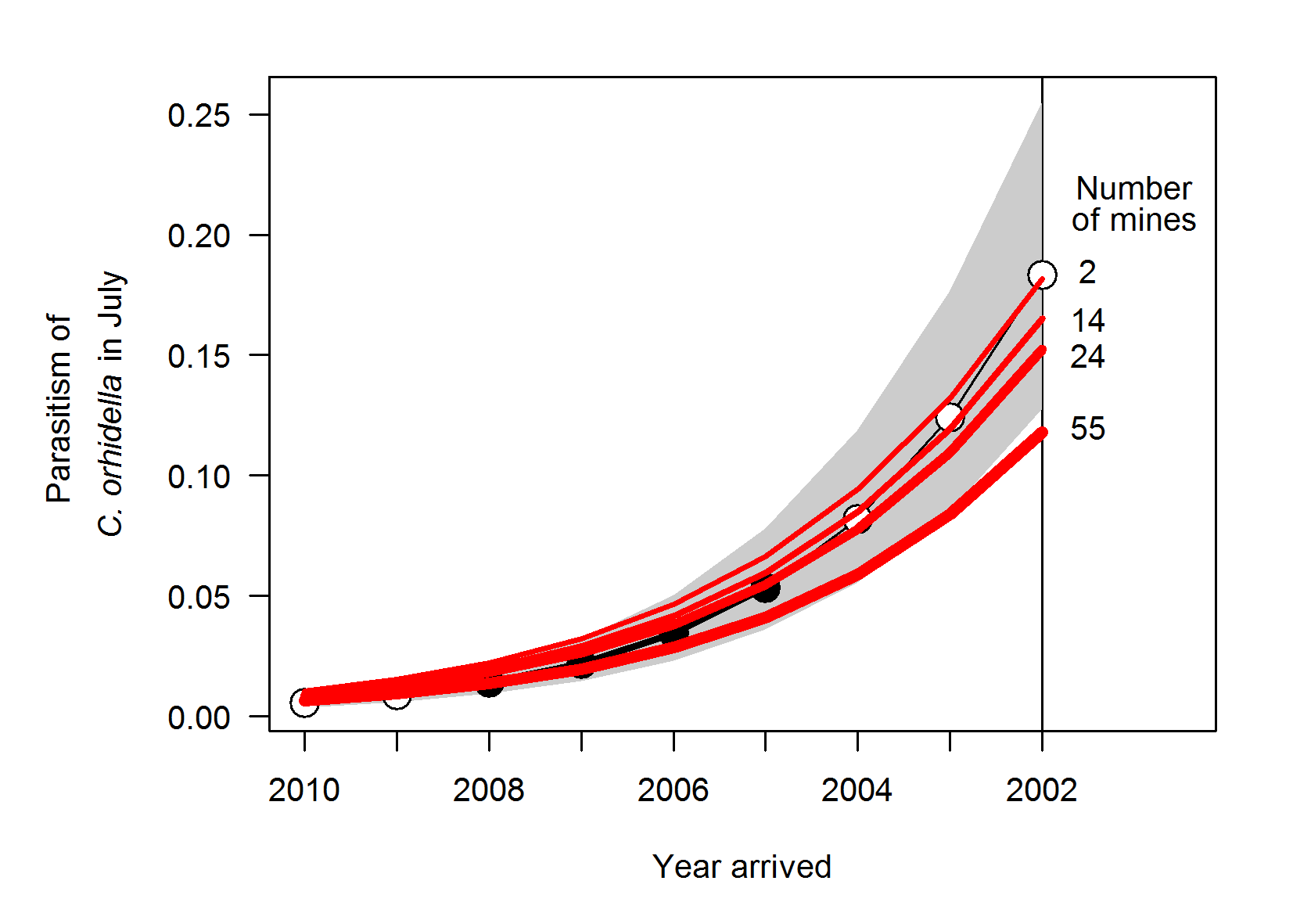

Supplement: Figure S3 — The effect of the number of mines on the estimated rate of parasitism. The results of the simple model (Fig. 2c) are overlaid with estimates from the full model for different numbers of mines, according to their distribution in the dataset (from top to bottom): 2 mines (5th percentile), 14 mines (median), 24 mines (75th percentile) and 55 mines (95th percentile). (TIF) [file pone.0086226.s003.tif]

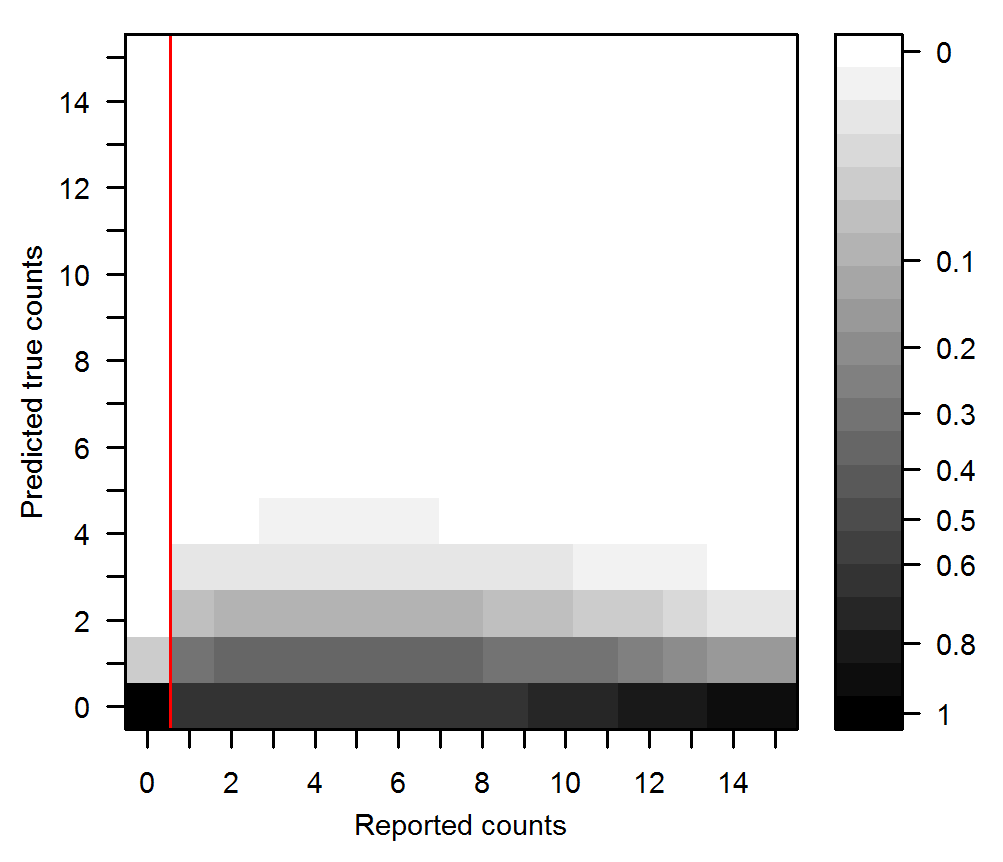

Supplement: Figure S4 — Modeled minimum number of parasitism events from reported counts of parasitoids. This model is the repeat of Fig. S3, but with the minimum number of parasitism events as defined in Methods S1. (TIF) [file pone.0086226.s004.tif]
