# Supplementary material for: The Success of the Horse-Chestnut Leaf-Miner, Cameraria ohridella, in the UK Revealed with Hypothesis-Led Citizen Science
Source: PLoS One. 2014 Jan 22;9(1):e86226. doi: 10.1371/journal.pone.0086226 (PMC3899221; doi:10.1371/journal.pone.0086226)
Supplement: Methods S1 — Measuring parasitism and taking account of uncertainty in the count data. (DOCX) [file pone.0086226.s005.docx]

### Supplementary Methods S1: measuring parasitism and taking account of uncertainty in the count data.

Parasitism of leaf-mining insects can be calculated in different ways. In the main paper we presented parasitism as: (total number of parasitoids) / (number of parasitoids plus *C. orhidella* adults). In using this definition, we assumed that each single parasitoid emerged from a single moth host, and we assumed that every non-parasitised caterpillar or parasitoid emerged as an adult. (We note that this assumption is often made in field studies of parasitoids, but is rarely explicitly stated.) We specifically tested the robustness of our results to these assumptions.

The first assumption that we considered was that all the emerging parasitoids were solitary. However, some parasitoids are gregarious and *Minotetrastichus frontalis*, which comprised about 50% of the parasitoids reared from in Britain in a related study [28], is the only potentially gregarious parasitoid of *C. ohridella* recorded in Europe [20]. 25% of the samples with *M. frontalis* in [28] had more than one *M. frontalis* (although because there were many hosts per sample, this does not prove that parasitoids were gregarious in these samples). Therefore, an alternative definition of parasitism is to consider the total number of parasitism events, rather than the total number of parasitoids. Practically this could be done by isolating the *C. orhidella* pupae individually, but this was impractical for a citizen science project. As an alternative definition of parasitism, we considered the ‘minimum number of parasitism events’, i.e. in which we made the conservative assumption that multiple *M. frontalis* per sample emerged from a single host and all other parasitoids were solitary. We identified all the parasitoids in the retained samples [28] and then we re-ran the zero-inflated Poisson models to estimate the relationship between the ‘minimum number of parasitism events’ and the observed number of parasitoids (Fig. S4). We then used this model to create distributions from which to sample the expected, true number of parasitism events, and ran the logistic regression as for the main analysis (see Methods).

The second assumption that we considered was that all live insects emerged. We are aware that, because of practical constraints, our instructions were to only rear insects for two weeks (starting between 3 and 9 July and finishing between 19 and 23 July 2010) which would have resulted in under-recording the potential number of insects that may have emerged. Local conditions may also have resulted in poor survival of larval moths and/or parasitoids. We tested the robustness of our conclusions by relating the number of parasitoids (or minimum number of parasitism events) to the number of leaf-mines, which represents the maximum potential number of hosts.

Thirdly, although we validated the counts of parasitoids and propagated the uncertainty into the final analyses (see Methods), we also considered the naïve assumption that all of the data were accurate and so did not require correction.

In testing our results to these three types of potential error, we found very little variation in the magnitude or significance of the effect of the length of time that *C. orhidella* had been present, but the actual estimated rate of parasitism did vary according to the assumptions that were being made (Table S1). Our bias-corrected data suggested rates of parasitism of between 1.3 and 5.1% after *C. orhidella* had been present for 6 years (depending on the assumptions made), which is broadly similar to previously published estimates of the true rate of parasitism, e.g. [20].
