# Supplementary material for: The Success of the Horse-Chestnut Leaf-Miner, Cameraria ohridella, in the UK Revealed with Hypothesis-Led Citizen Science
Source: PLoS One. 2014 Jan 22;9(1):e86226. doi: 10.1371/journal.pone.0086226 (PMC3899221; doi:10.1371/journal.pone.0086226)
Supplement: Table S1 — Results of the best model from Table 2 (log-linear segmented regression with the break point being at two years and assuming a modeled distribution with two generations per year) with additional effects of location (eastings and northings). (DOCX) [file pone.0086226.s006.docx]

| Model | AIC | ΔAIC |
| --- | --- | --- |
| Original model | 2426.5 | 46.2 |
| +eastings | 2400.9 | 20.6 |
| +northings | 2419.1 | 38.8 |
| +eastings+northings | 2380.3 | 0.0 |
| +eastings+northings+their interaction | 2382.3 | 2.0 |

**Table S1.** Results of the best model from Table 2 (log-linear segmented regression with the break point being at two years and assuming a modeled distribution with two generations per year) with additional effects of location (eastings and northings). This shows the importance of location, with the effects being significant (P<0.001 for both in the best-ranked model) with the effect of northing being negative and easting being positive, thus being confounded with the length of time that the most had been present.
