# Supplementary material for: The Success of the Horse-Chestnut Leaf-Miner, Cameraria ohridella, in the UK Revealed with Hypothesis-Led Citizen Science
Source: PLoS One. 2014 Jan 22;9(1):e86226. doi: 10.1371/journal.pone.0086226 (PMC3899221; doi:10.1371/journal.pone.0086226)
Supplement: Table S2 — Effect size of the length of time that the C. orhidella had been present when considering different numerators and denominators in the definition of parasitism, and whether the data were adjusted to correct for bias in the reported data. (DOCX) [file pone.0086226.s007.docx]

| Numerator | Denominator | Adj. | Intercept | Effect | P | Parasitism |
| --- | --- | --- | --- | --- | --- | --- |
|  |  |  | (SE) | size (SE) |  | at 6 years (%) |
| All parasitoids | Emergents | None | -5.17 | 0.50 | <0.001 | 10.2 |
|  |  |  | (0.64) | (0.14) |  |  |
| All parasitoids | Emergents | All | -5.57 | 0.44 | 0.012 | 5.1 |
|  |  |  | (0.77) | (0.16) |  |  |
| All parasitoids | Leaf mines | None | -6.24 | 0.43 | 0.005 | 2.5 |
|  |  |  | (0.72) | (0.15) |  |  |
| All parasitoids | Leaf mines | All | -6.83 | 0.44 | 0.007 | 1.4 |
|  |  |  | (0.72) | (0.15) |  |  |
| Min. parasitism events | Emergents | All | -5.68 | 0.46 | 0.005 | 5.0 |
|  |  |  | (0.72) | (0.15) |  |  |
| Min. parasitism events | Leaf mines | All | -6.88 | 0.42 | 0.004 | 1.3 |
|  |  |  | (0.65) | (0.14) |  |  |

**Table S2.** Effect size of the length of time that the *C. orhidella* had been present when considering different numerators and denominators in the definition of parasitism, and whether the data were adjusted to correct for bias in the reported data. The ‘minimum number of parasitism event’ was calculated as described in the text. ‘Emergents’ is the total number of reported insects (i.e. reported adult moths + parasitoids). ‘Adj.’ = Adjustment, which was made on the basis of the results of the zero-inflated Poisson models (see Methods). Where there was bias-correction, the standard error (SE) was obtained by multiple imputation of analysis of the bootstrapped data (see Methods). ‘Parasitism at 6 years’ is estimated parasitism for sites where *C. ohridella* arrived in 2005, and is calculated as: logit^-1^(Intercept+ (6×Effect size)).
